# Considerations for Addressing Trauma in Muslim Communities

**DOI:** 10.1001/jamanetworkopen.2024.29605

**Published:** 2024-08-01

**Authors:** Manasi Kumar, Keng Yen Huang

**Affiliations:** Institute for Excellence in Health Equity, New York University (NYU) Grossman School of Medicine, New York; Center for Early Childhood Health & Development (CEHD), Department of Population Health, NYU Grossman School of Medicine, New York

Although progress has been made in providing cost-effective strategies to implement mental health interventions in the field of global mental health research, progress on successfully adapting interventions to diverse cultures, countries, and population contexts is slow.^1^ It remains unclear whether our current approach to implementing mental health interventions needs to be adjusted based on the unique vulnerabilities and exposures of specific patient groups across geographies, keeping in mind the following concerns: (1) how these vulnerabilities might impact the effectiveness of our interventions; (2) what resources are necessary for successful implementation; and (3) the essential components of a support system to ensure successful implementation of mental health interventions. Providing mental health interventions in non-Western geographies and populations merits further attention.^2^ Zoellner et al^3^ present a fascinating adaptation of a multipronged intervention that addresses mental health needs of Somali refugees living in 2 US states of Ohio and Washington. The intervention focuses on Muslim refugees and immigrants who met the criterion of posttraumatic stress disorder and were then offered an intervention delivered by lay health workers in conjunction with religious leaders. The study contributes to new knowledge and advancement in the field of mental health therapeutics as the previously developed Islamic Trauma Healing intervention was highly effective at obtaining remission from trauma, depression, and anxiety at 3 months postintervention although it did not yield significant improvements in somatic symptoms. The intervention was well-received and developed with a large team with community engagement and consultation. Our commentary attempts to highlight how reporting of adaptation process, formulating theory of change around the intervention, and reflecting on implementation context would provide additional insights.

While developing an effective multipronged intervention, we believe additional efforts have to be made to deepen cultural adaptation methodology embedded within an intervention framework, generating new knowledge about strategies adopted toward mental health framework implementation. We offer suggestions that researchers from the psychiatry and mental health field may wish to consider when carrying out cultural adaptations of mental health interventions for non-Western populations.

## Prioritize Reporting Adaptation Process and Cultural Perspectives in an Integrated Manner

### Steps Associated With Deep Cultural Adaptation

Most trauma intervention research based in lower-and-middle income settings (LMICs) and those focusing especially on underserved communities in high income countries (HICs) (as conducted by Zoellner et al^3^) do not describe adaptation process with regard to what aspects of cultural processes are studied and leveraged toward social norms and/or behavioral change. Religion here is important but only one of the several elements of cultural identity. To better understand the role of culture and religion in advancing mental health outcomes, how faith and religious beliefs are conceptualized in the intervention, and how these become a conduit toward specific community and individual level mental health change need to be considered. Further research is warranted to explore how the cultural psyche undergoes transformation during the trauma recovery process within specific populations and consider ways in which communities might want to reflect on this change process in their own lexicon and present lived experience in some depth. Unpacking these cultural nuances can help unravel the mechanisms underlying trauma healing and recovery after complex humanitarian crises leading toward more sensitive and effective interventions being developed for affected individuals and communities.

### Etic and Emic Approaches in the Intervention Adaptation Process

An *etic* approach, assumes that behavioral constructs (ie, concepts, methods, measures) studied in one culture have significance in all other cultures, while an *emic* approach describes the study of cultural norms, values, and perspectives that are specific to a group of people or within a culture.^4^ Most global mental health intervention research focuses on the etic (cross-cultural) aspect while analyzing characteristics and behavior across different cultural groups, with an interest in broad variations and testing universal theory of posttraumatic stress disorder. By contrast, the field of cultural psychology lends itself to an emic perspective, which allows the researchers and interventionists to delve into an understanding of how that culture would redress trauma-associated manifestations, reality of refugeehood, reality of becoming an immigrant in another cultural setting, and acculturation process keeping an idiographic perspective in mind.

The Zoellner and colleagues^3^ study is largely based on an etic conceptualization of cultural adaptations, in harmony with how most global mental health research in LMICs and high adversity immigrant or refugee populations is conducted. The formative inquiries published earlier by Zoellner and colleagues^5-7^ provide the rationale and approach adopted in the *Islamic Healing Intervention*. The etic perspective has advantages of cross-checking constructs and their applicability, while at the same time, it is disadvantageous as it does not let indigenous voices and traditional beliefs, systems of practice, newer lexicons, or new theoretical perspectives emerge independently. In this regard, we advocate for researchers, practitioners, academic journals (and even funders) to encourage use of emic conceptualizations of mental health intervention research to unpack trauma and other complex exposures that refugee and immigrant communities experience in today’s world. Only that sort of conceptualization of subjective experience and contextualization can advance understanding of socio-cultural, religious, political, and psychological identities and how and why these are fragmented and disturbed in the aftermath of trauma. An emic perspective helps in understanding innate resources and strengths that can contribute to a more resilient self and community development. Such an approach through its exploration of specific internal resources within individuals and groups could provide novel ways of conceptualizing posttraumatic growth, coping mechanisms, bolstering of self and social bonds differently. In this the emic conceptualization borrows from both an asset-based approach and a traditional deficit-based trauma conceptualization.

## Theories of Change Around Trauma Intervention

Most behavioral theories of posttraumatic stress disorder and trauma intervention are based on Western theoretical perspectives and systems of disease classification and symptom definition.^7^ It is not clear how the typical trauma intervention theory aligns with Islamic beliefs and faith-based practices. Likewise, Zoellner et al^3^ do not clarify whether somatic symptoms are part of depressive symptomatology in Muslim populations, for example, those exposed to traumatic events and whether they should be monitored as a key outcome of depression. A conceptual model explaining cultural perspective of traumatic stress and of recovery and healing process that considers core values of Islam as a religion, focuses on faith healing traditions, symptom expression differences, and then applies this culturally adapted framework to guide the intervention design and testing is likely to have greater impacts in Muslim and similarly underinvestigated refugee populations. This is particularly notable given the ongoing upheavals experienced in several Muslim geographies globally.

## Intervention Implementation Context and Strategies to Provide Mental Health Support in Diverse Ethnic, Cultural, or Religious Groups

There is so much to be learned around how to provide mental health interventions to Muslim or other non-Western religious believers or those in diverse cultural settings. Providing more contextual information for intervention implementation for example about Islamic faith context (eg, structural settings, gender norms, ethnic identity, ethnic or granular religious practices) can help disseminate knowledge and provide a frame of reference to other researchers and practitioners for setting up culturally appropriate and responsive interventions. Most of the participants in the Zoellner study^3^ were women who routinely received traumatic relocations and continue to have marginalized identities in non-Western societies, be it in LMICs or HICs. No data on the make-up of the implementors was shared, so we cannot assess whether their identities affected the study outcomes. In addition, little attention was paid to gender, ethnic identity, or cultural beliefs and practices surrounding illness and healing practices common within the study cohort. Notably, the same authors published earlier studies presenting data on cultural adaptation processes, although these were largely epidemiologic inquiries.^8,9^

We appreciate the work carried out by Zoellner and colleagues^3^ in extending an innovative trauma-informed intervention. While the findings of Zoellner and colleagues^3^ bring to the fore the importance of timely faith-based intervention delivered within faith-based settings, we want to inspire global mental health researchers to go the extra mile to report on the processes of adaptation, extend knowledge of the implementation context, resources needed for implementation as well as deepen the experiential understanding of specific trauma-associated mechanisms^10^ that are being targeted and develop a theory of change perspective to demonstrate how trauma healing and recovery is being visualized through the culturally adapted intervention.
